# Supplementary material for: Radiation combined with macrophage depletion promotes adaptive immunity and potentiates checkpoint blockade
Source: EMBO Mol Med. 2018 Nov 15;10(12):e9342. doi: 10.15252/emmm.201809342 (PMC6284388; doi:10.15252/emmm.201809342)
Supplement: Supplementary file 1 — Appendix [file EMMM-10-e9342-s001.pdf]

Appendix – Table of Contents

Content	Page number
Appendix Figure S1	2
Appendix Figure S2	4
Appendix Figure S3	6
Appendix Table S1	8
Appendix Table S2	8
Appendix Table S3	9-11

Appendix Figure S1

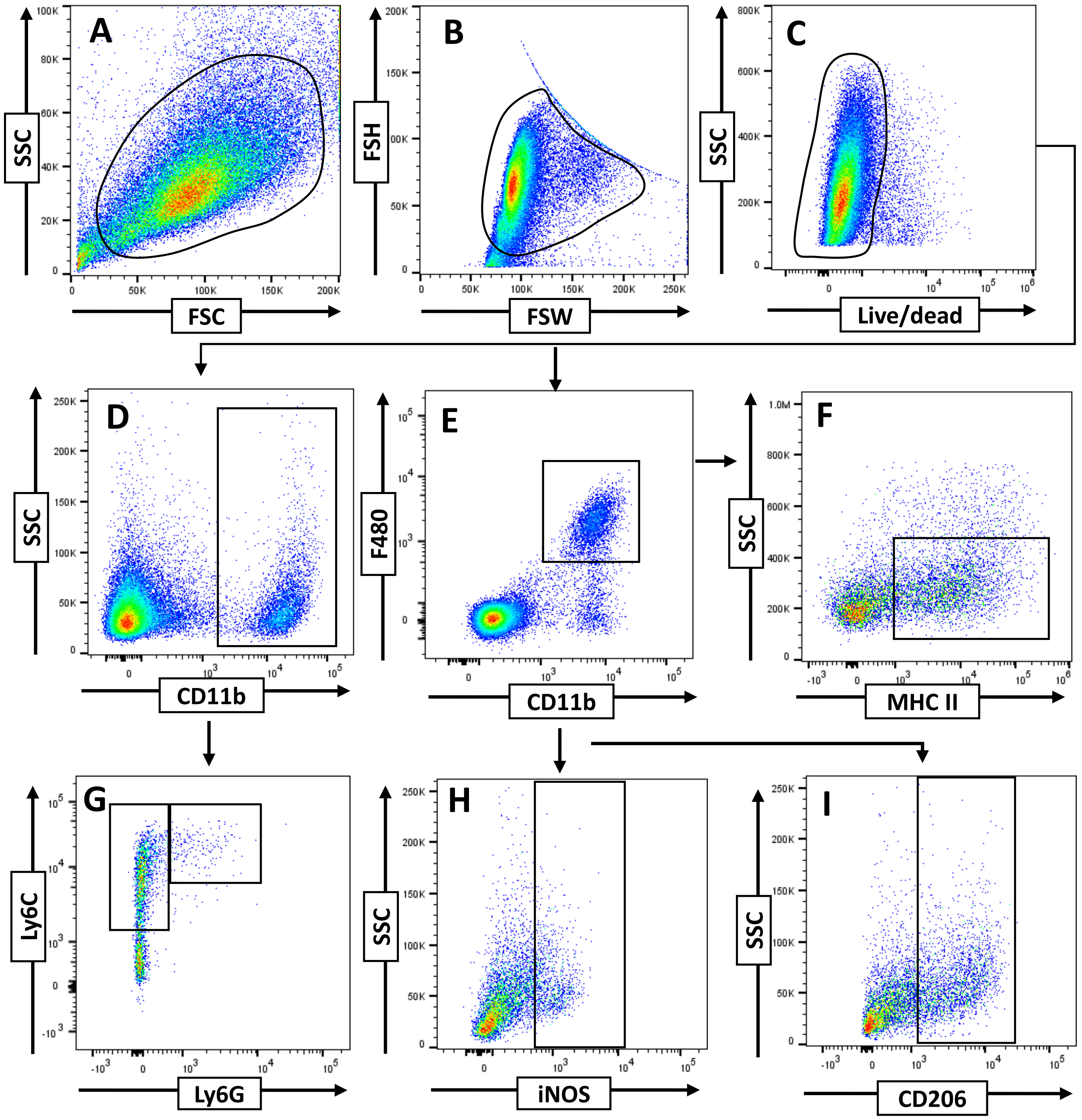

Lymphocyte flow cytometry gating strategy

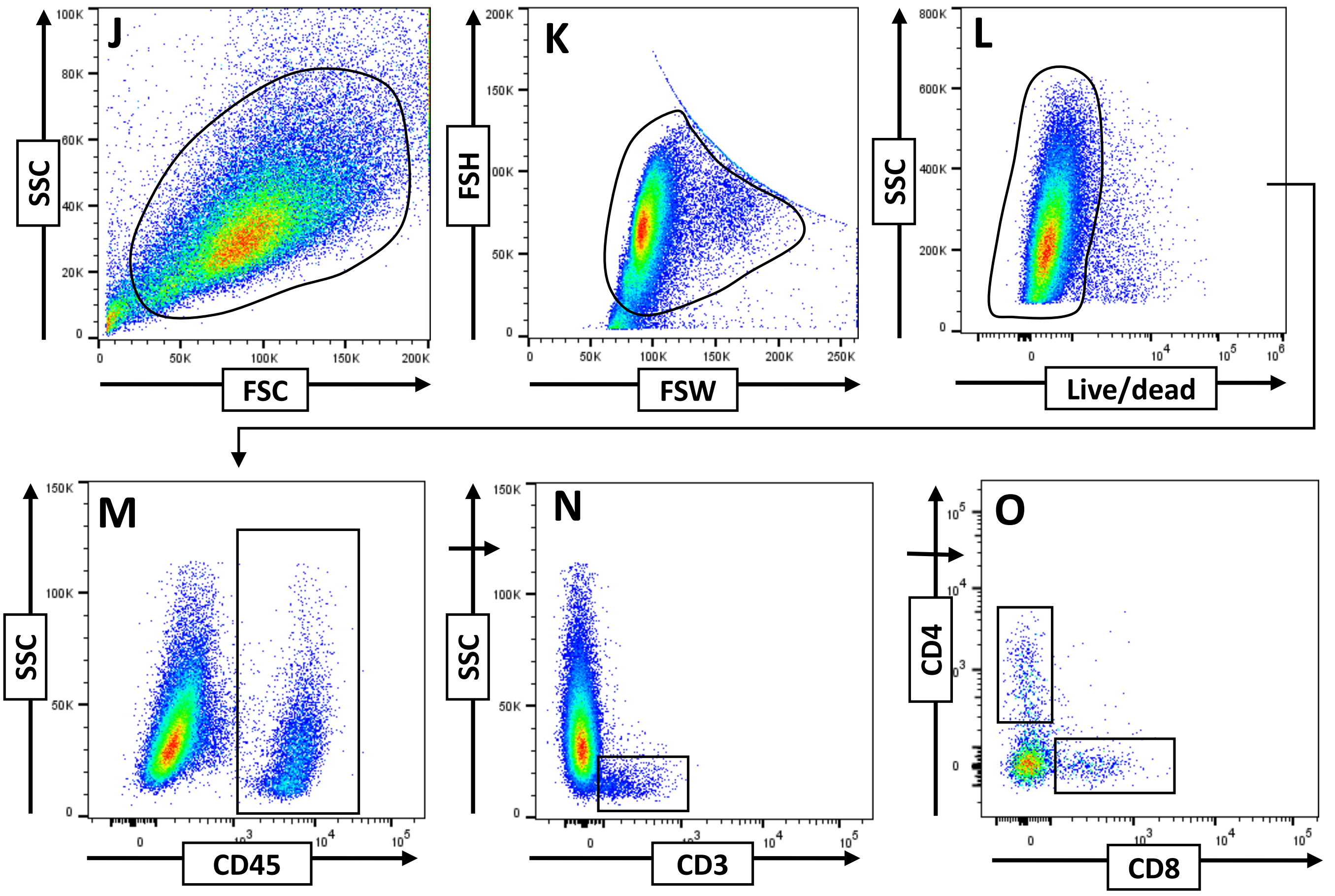

Appendix Figure S1– Flow cytometry gating strategy for myeloid and lymphocytic populations. Representative flow cytometry dot plots indicating the gating strategy for identification of myeloid and lymphocyte populations. A-C – Gates were used to exclude debris (A), doublets (B) and dead cells (C). CD11b+ cells (D) were gated for subsequent analysis of Ly6C (monocytic MDSCs) vs Ly6G (granulocytic MDSCs) (G). CD11b+F480+ macrophages (E) were gated, and MHC II (F) iNOS (H) and CD206 (I) expression measured. For lymphocytes, CD45+ (M) were subsequently analysed for CD3 (N) and CD4 or CD8 (O).

Appendix Figure S2

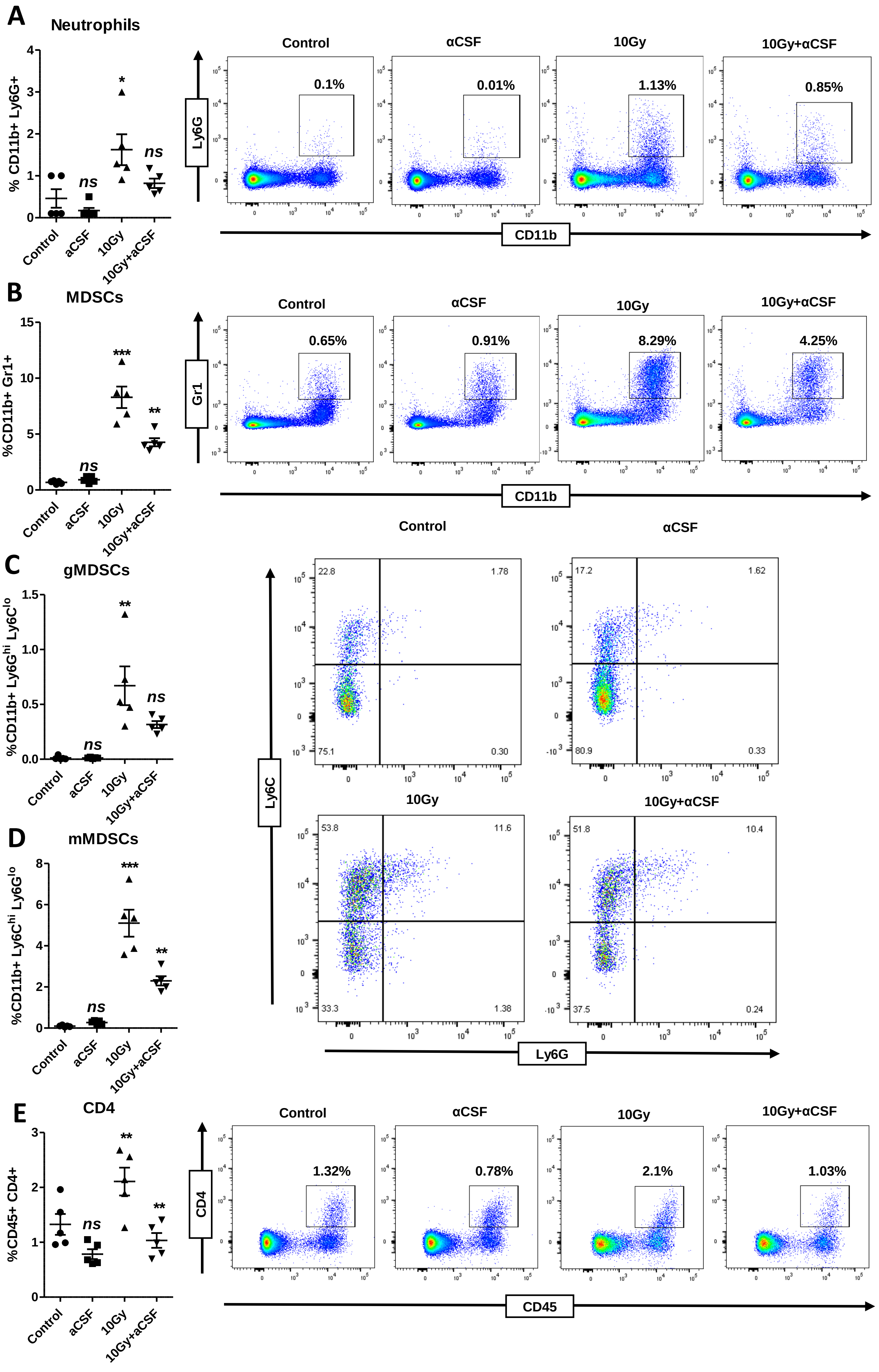

Appendix Figure S2– aCSF combined with radiation is associated with variable changes in myeloid and lymphocytic populations in MC38 tumours. Mice bearing MC38 tumours received treatments as indicated**.** For irradiation groups, tumours were harvested 5 days following radiation. Myeloid populations included neutrophils (CD11b+ Ly6G+, A), myeloid derived suppressor cells (CD11b+ Gr1+, B), granulocytic MDSCs (Ly6G^{hi} Ly6C^{lo}, C), monocytic MDSCs (Ly6C^{hi} Ly6G^{lo}, D) and CD4 T cells (CD45+ CD4+, E).

Appendix Figure S3

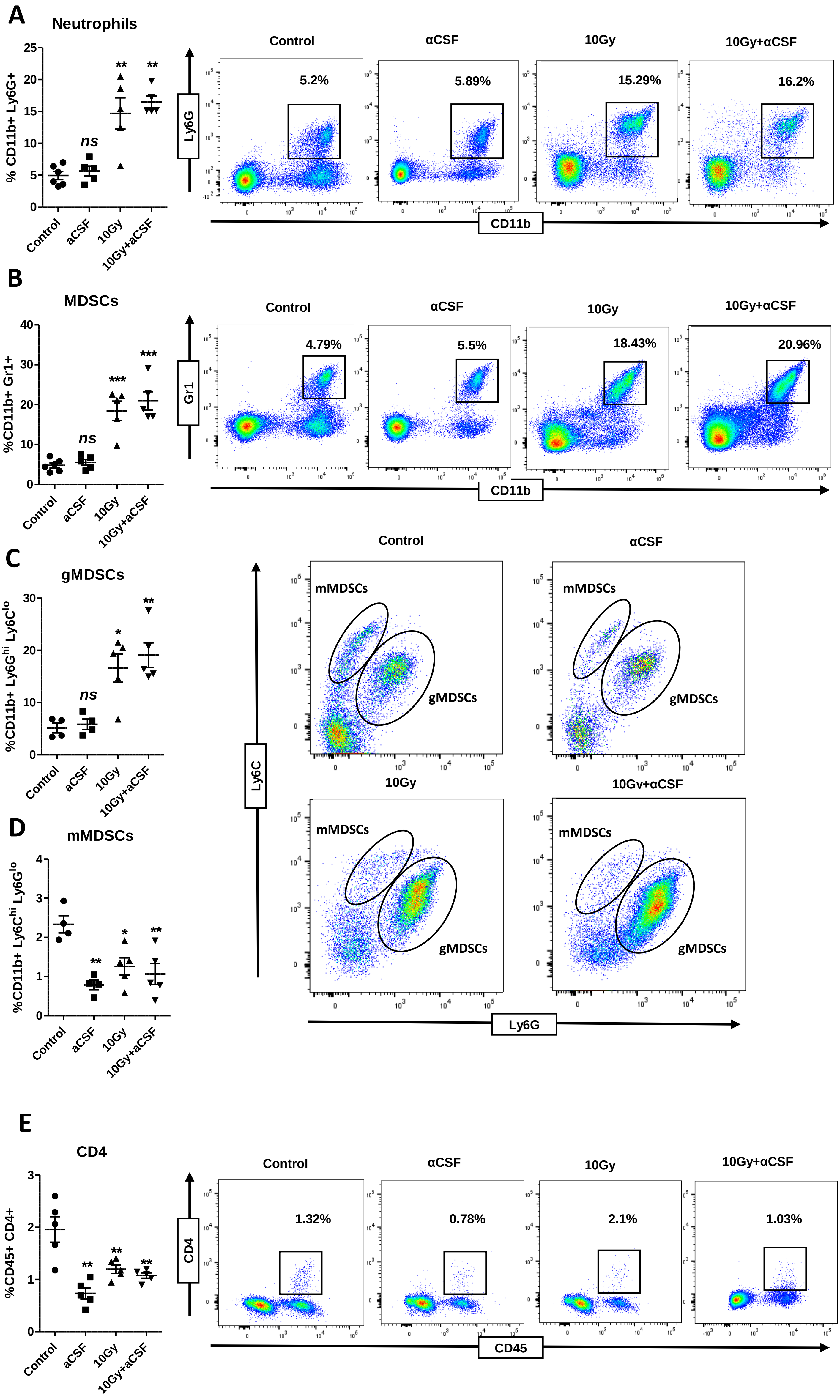

Appendix Figure S3 – aCSF combined with radiation is associated with variable changes in myeloid and lymphocyte populations in KPC tumours. Tumours were harvested from mice bearing KPC tumours receiving treatment as indicated. For irradiation groups, tumours were harvested 5 days following radiation. Myeloid populations included neutrophils (CD11b+ Ly6G+, A), myeloid derived suppressor cells (CD11b+ Gr1+, B), granulocytic MDSCs (Ly6G^{hi} Ly6C^{lo}, C), monocytic MDSCs (Ly6C^{hi} Ly6G^{lo}, D) and CD4 T cells (CD45+ CD4+, E).

Appendix Table S1

Antibody	Supplier	Catalogue number	Volume/10^6 cells
Cell viability dye	Ebiosciences	65-0867-18	1µL
CD11b PeCy7	Ebiosciences	25-0112-81	0.625µL
CD8a PeCy7	Ebiosciences	25-0081-82	1.0µL
CD4 APC	Ebiosciences	17-0041-82	1.25µL
CD3 APC-Cy7	Ebiosciences	47-0031-82	0.5µL
F480 BV 421	Biolegend	123132	1.0µL
MHC II APC	Biolegend	17-5321-82	1.0µL
CD11c PE	Ebiosciences	12-0114-81	0.2µL
NK 1.1 APC Cy-7	Biolegend	108724	0.5µL
Granzyme APC	ThermoFisher	25-8898-82	2.5µL
Interferon-γ APC	Ebiosciences	17-7311-81	1.0µL
Ki67 PE	Ebiosciences	12-5698-80	0.2µL
PD-1 BV 421	Biolegend	135221	1.0µL
PDL-1 BV 421	Biolegend	124315	1.0µL
Ly6C APC Cy-7	Biolegend	128026	0.5µL
Ly6G-Gr1 BV 510	Biolegend	108438	1.0µL
CD206 PerCP Cy 5.5	Biolegend	141715	1.0µL
iNOS PE	Santa Cruz	Sc-7271	0.2µL
CD45 BV 510	Biolegend	103138	0.625µL

Appendix Table S2

Gene	Forward primer	Reverse primer
B-actin	GATGTATGAAGGCTTTGGTC	TGTGCACTTTTATTGGTCTC
CSF-1	TAGAAAGGATTCTATGCTGGG	CTCTTTGGTTGAGAGTCTAAG
iNOS	CATCAACCAGTATTATGGCTC	TTTCCTTTGTTACAGCTTCC
Il12	GAAGACATCGATCATGAAGAC	CTCTTGTTGTGGAAGAAGTC
TNF-α	GTAGCCCACGTCGTAGCAAA	ACAAGGTACAACCCATCGGC
Il1α	AACCCATGATCTGGAAGAGACC	TGGTGCTGAGATAGTGTTTGTCC
Il1ß	CAGGCAGTATCACTCATTGTGG	GTGCAGTTGTCTAATGGGAACG
Arginase	CTGACCTATGTGTCATTTGG	CATCTGGGAACTTTCCTTTC
Il6	ACAAAGCCAGAGTCCTTCAGAGA	CTGTTAGGAGAGCATTGGAAATTG
Ym1	TCTGGTGAAGGAAATGCGTAAA	GCAGCCTTGGAATGTCTTTCTC
CCL2	CAAGATGATCCCAATGAGTAG	TTGGTGACAAAAACTACAGC
Il10	CAGGACTTTAAGGGTTACTTG	ATTTTCACAGGGGAGAAATC

Figure	Group	n	P value	Test
1B	MC38 10Gy 24hrs	3	0.0224	Kruskal-Wallis
1B	KPC 10Gy 24hrs	3	0.0065	Kruskal-Wallis
1B	KPC 10Gy 48hrs	3	<0.0001	Kruskal-Wallis
1B	KPC 10Gy 72hrs	3	0.00021	Kruskal-Wallis
1C	MC38 10Gy	3	0.0045	Mann Witney
1C	KPC 10Gy	3	0.0063	Mann Witney
1D	MC38 10Gy D3	4	0.044	Kruskal-Wallis
1D	KPC 0Gy	4	0.032	Kruskal-Wallis
1D	KPC 10Gy D7	4	0.0019	Kruskal-Wallis
1E	D1	6	0.0046	Kruskal-Wallis
1E	D3	6	0.0034	Kruskal-Wallis
1E	D5	6	0.0055	Kruskal-Wallis
1E	D10	6	0.002	Kruskal-Wallis
1E	D14	6	0.0023	Kruskal-Wallis
1F	D5	6	0.00026	Kruskal-Wallis
1I	Macrophages	3	0.0085	Unpaired t test
1I	Neutrophils	3	0.021	Unpaired t test
1I	MDSCs	3	0.00015	Unpaired t test
1I	CD4	3	0.033	Unpaired t test
2A	MC38 iNOS	6	0.008	Mann Witney
2A	KPC iNOS	6	0.0006	Mann Witney
2B	MC38 CD206	6	0.0079	Mann Witney
2C	MC38	6	<0.0001	Mann Witney
2C	KPC	6	0.0045	Mann Witney
2D	MC38	6	0.0099	Mann Witney
2D	KPC	6	0.0018	Mann Witney
2E	MC38	6	0.01	Mann Witney
2E	KPC	6	0.00043	Mann Witney
2F	MC38	6	0.0079	Mann Witney
2G	il1ra	3	0.034	Mann Witney
2G	Il1b	3	0.0045	Mann Witney
2G	Arg	3	0.00026	Mann Witney
2G	ccl2	3	0.0085	Mann Witney
2G	il10	3	0.0014	Mann Witney
2H	NOS2	3	0.043	Mann Witney
2H	Arg	3	0.0046	Mann Witney
2H	Ym1	3	0.0033	Mann Witney
2I	No XRT	3	0.0004	Kruskal-Wallis
2I	XRT	3	0.029	Kruskal-Wallis
3B	aCSF	6	<0.0001	1-way ANOVA
3B	10Gy + aCSF	6	<0.0001	1-way ANOVA
3C	aCSF	6	0.0028	1-way ANOVA
3C	10Gy + aCSF	6	0.00012	1-way ANOVA
3D	10Gy + IgG	6	<0.0001	1-way ANOVA
3D	10Gy + aCSF	6	0.00025	1-way ANOVA

Figure	Group	n	P value	Test
3E	10Gy + IgG	6	0.00016	1-way ANOVA
3E	10Gy + aCSF	6	<0.0001	1-way ANOVA
3F	MC38 aCSF	6	0.00027	1-way ANOVA
3F	MC38 10Gy + aCSF	6	0.00034	1-way ANOVA
3F	KPC aCSF	6	0.045	1-way ANOVA
3F	KPC 10Gy + aCSF	6	0.0049	1-way ANOVA
3G	MC38 10Gy	6	0.033	1-way ANOVA
3G	MC38 10Gy + aCSF	6	0.012	1-way ANOVA
3H	Nos2	3	0.00023	Mann Witney
3H	Il12	3	0.044	Mann Witney
3H	Tnfa	3	0.00046	Mann Witney
3H	il1a	3	0.0001	Mann Witney
3H	Arg	3	<0.0001	Mann Witney
3H	ccl2	3	0.00069	Mann Witney
3H	il10	3	0.00081	Mann Witney
3I	Arg	3	0.00079	Mann Witney
3I	Il10	3	0.00068	Mann Witney
3I	il6	3	0.0005	Mann Witney
3I	Nos2	3	0.00029	Mann Witney
3I	Tnfa	3	0.00061	Mann Witney
3I	il1a	3	<0.0001	Mann Witney
4A	aCSF	6	0.00096	1-way ANOVA
4A	10Gy + aCSF	6	0.00081	1-way ANOVA
4C	aCSF	6	0.00047	Kruskal-Wallis
4C	10Gy	6	0.00036	Kruskal-Wallis
4D	10Gy	6	0.012	Kruskal-Wallis
4D	10Gy + aCSF	6	0.032	Kruskal-Wallis
4E	aCSF	6	0.03	Kruskal-Wallis
4E	10Gy + aCSF	6	0.024	Kruskal-Wallis
4F	10Gy + aCSF	6	0.011	Kruskal-Wallis
4I	10Gy + aCD8	3	0.0025	1-way ANOVA
4I	10Gy + IgG	3	0.00096	1-way ANOVA
4I	10Gy + aCSF	3	0.00075	1-way ANOVA
4J	10Gy + aCD8	3	0.032	1-way ANOVA
4J	10Gy + aCSF	3	0.00026	1-way ANOVA
4J	10Gy + aCD8	3	0.00055	1-way ANOVA
4K	aCD8	5	<0.0001	Unpaired t test
4K	10Gy + aCD8	5	<0.0001	Unpaired t test
4L	10Gy + IgG	6	0.00036	1-way ANOVA
4L	10Gy + aCSF	6	0.00085	1-way ANOVA
5A	10Gy + irradiated	3	0.02	Kruskal-Wallis
5C	10Gy	3	<0.0001	Mann Witney
5E	10Gy	3	<0.0001	Unpaired t test
5F	10Gy	3	<0.0001	Unpaired t test
5J	Primary	5	0.00038	Unpaired t test

Figure	Group	n	P value	Test
5L	Primary	5	0.005	Unpaired t test
5M	Primary	5	0.00033	Unpaired t test
5N	aCSF vs 10Gy+aCSF	5	0.0041	Kruskal-Wallis
5N	10Gy vs 10Gy+aCSF	5	0.0001	Kruskal-Wallis
5O	aCSF	5	0.00022	Kruskal-Wallis
6A	10Gy	3	0.011	Mann Witney
6B	10Gy	3	0.042	Mann Witney
6C	10Gy	5	0.00046	Unpaired t test
6D	10Gy	5	0.00049	Unpaired t test
6I	Primary	8	0.00027	Unpaired t test
6J	Primary	8	0.0056	Unpaired t test
EV2A	iNOS CTRL	3	0.0044	Kruskal-Wallis
EV2A	CD206 CTRL	3	0.001	Kruskal-Wallis
EV2B	CD206 CTRL	3	0.00022	Kruskal-Wallis
EV2B	CD206 10Gy	3	0.00012	Kruskal-Wallis
EV2E	10Gy	3	0.012	Kruskal-Wallis
EV2F	CTRL	3	0.02	Kruskal-Wallis
EV2F	10Gy	3	0.019	Kruskal-Wallis
EV4A	10Gy+aCSF	5	0.044	Unpaired t test
EV4B	10Gy+aCSF	5	0.03	Unpaired t test
EV5A	aCSF	5	<0.0001	Kruskal-Wallis
EV5A	10Gy	5	0.029	Kruskal-Wallis
EV5A	10Gy+aCSF	5	<0.0001	Kruskal-Wallis
EV5B	aCSF	5	0.0071	Kruskal-Wallis
EV5B	10Gy+aCSF	5	0.039	Kruskal-Wallis
