# Supplementary material for: Radiation combined with macrophage depletion promotes adaptive immunity and potentiates checkpoint blockade
Source: EMBO Mol Med. 2018 Nov 15;10(12):e9342. doi: 10.15252/emmm.201809342 (PMC6284388; doi:10.15252/emmm.201809342)
Supplement: Supplementary file 2 — Expanded View Figures PDF [file EMMM-10-e9342-s002.pdf]

Expanded View Figures

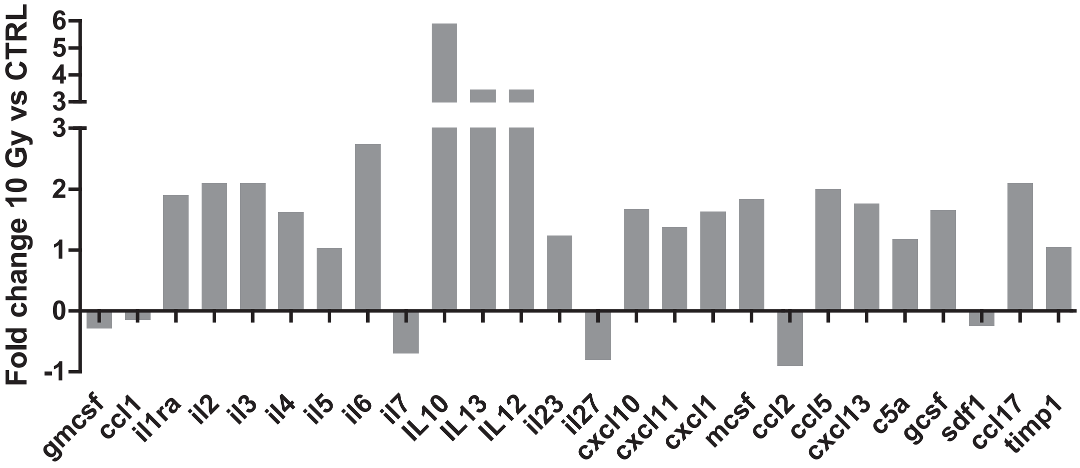

Figure EV1. Irradiation stimulates the secretion of CSF-1 by tumour cells.

Cytokine array analysis of conditioned media (CM) collected from MC38 cells 48 h after irradiation (10 Gy) compared with CM from naÿve MC38 cells. Data are presented as fold changes normalised to levels in unirradiated tumour cells.

Figure EV2. Bone marrow-derived macrophages (BMDMs) are polarised following co-culture with tumour cells.

A, B Flow cytometric analysis of iNOS and CD206 expression on wild-type (WT) BMDMs and BMDMs co-cultured with naïve or irradiated MC38 (A) and KPC (B) tumour cells. Representative histograms are also shown (bottom panel). Data are presented as mean ± SEM and analysed by Kruskal–Wallis test with Dunn's multiple comparisons test (n = 3/group).

C, D RNA was extracted from BMDMs co-cultured with MC38 (C) and KPC (D) tumours cells. Expression of selective inflammatory and immunosuppressive genes was analysed by RT–qPCR. Data shown are fold changes compared to WT. Data are presented as mean ± SEM and analysed by Kruskal–Wallis with Dunn's multiple comparisons test (n = 3/group).

E, F T-cell proliferation suppressive activity of BMDMs co-cultured with MC38 (E) and KPC (F) tumour cells. Data are presented as mean ± SEM and analysed by Kruskal–Wallis test with Dunn's multiple comparisons test. BMDMs were co-cultured for 72 h before being added at a 1:1 ratio with carboxyfluorescein succinimidyl ester (CFSE) labelled naïve murine $CD8^+$ T cells for 55 h. CFSE dilution was analysed by flow cytometry. Percentage of $CFSE^{lo}$ T cells were quantified and analysed by unpaired t-test. Experiments were repeated twice per cell line to ensure statistical concordance (n = 3).

Data information: $^*P < 0.05$, $^{**}P < 0.01$, $^{***}P < 0.001$.

© 2018 The Authors

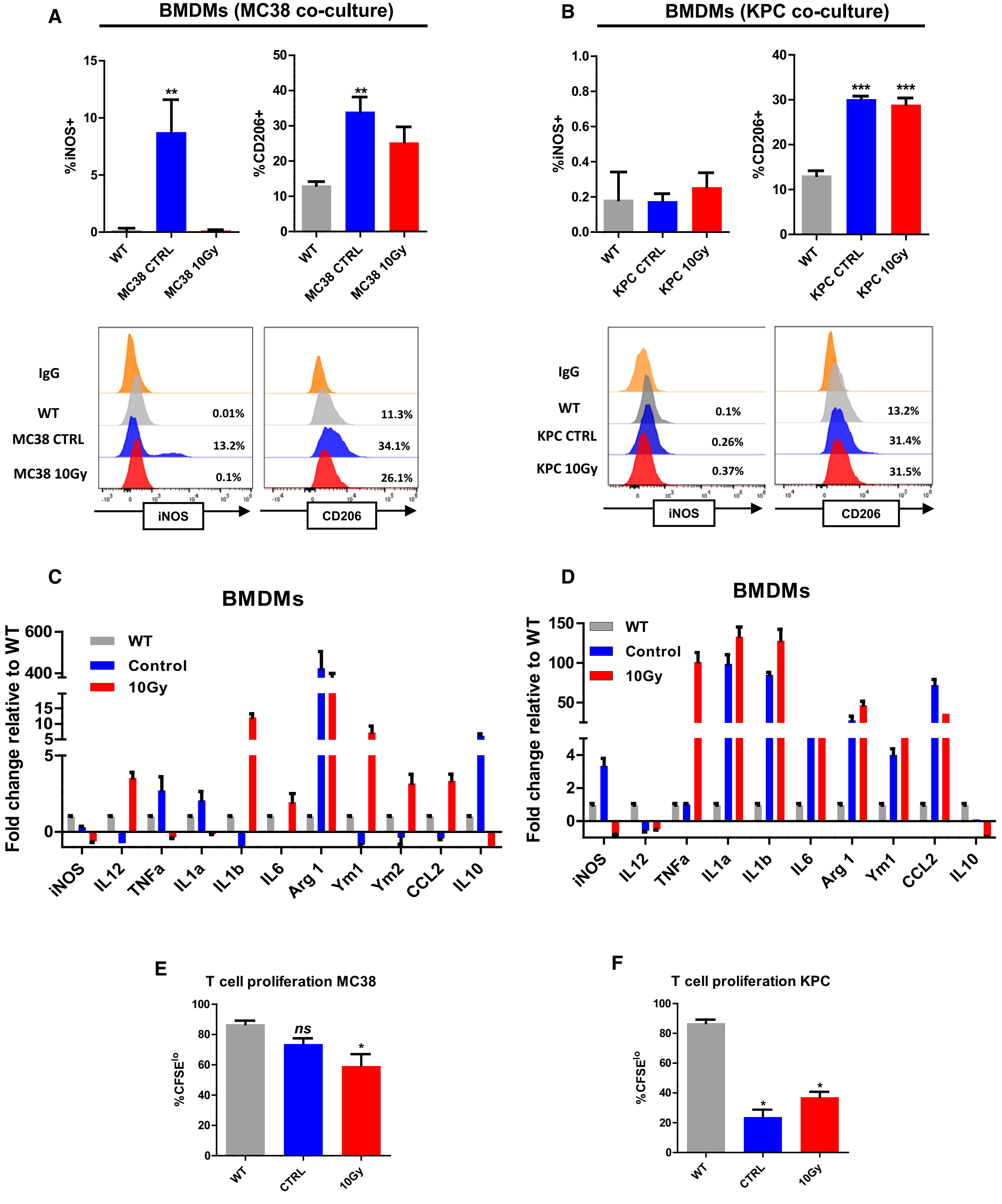

Figure EV2.

 © 2018 The Authors

Clonogenic survival +/- aCSF

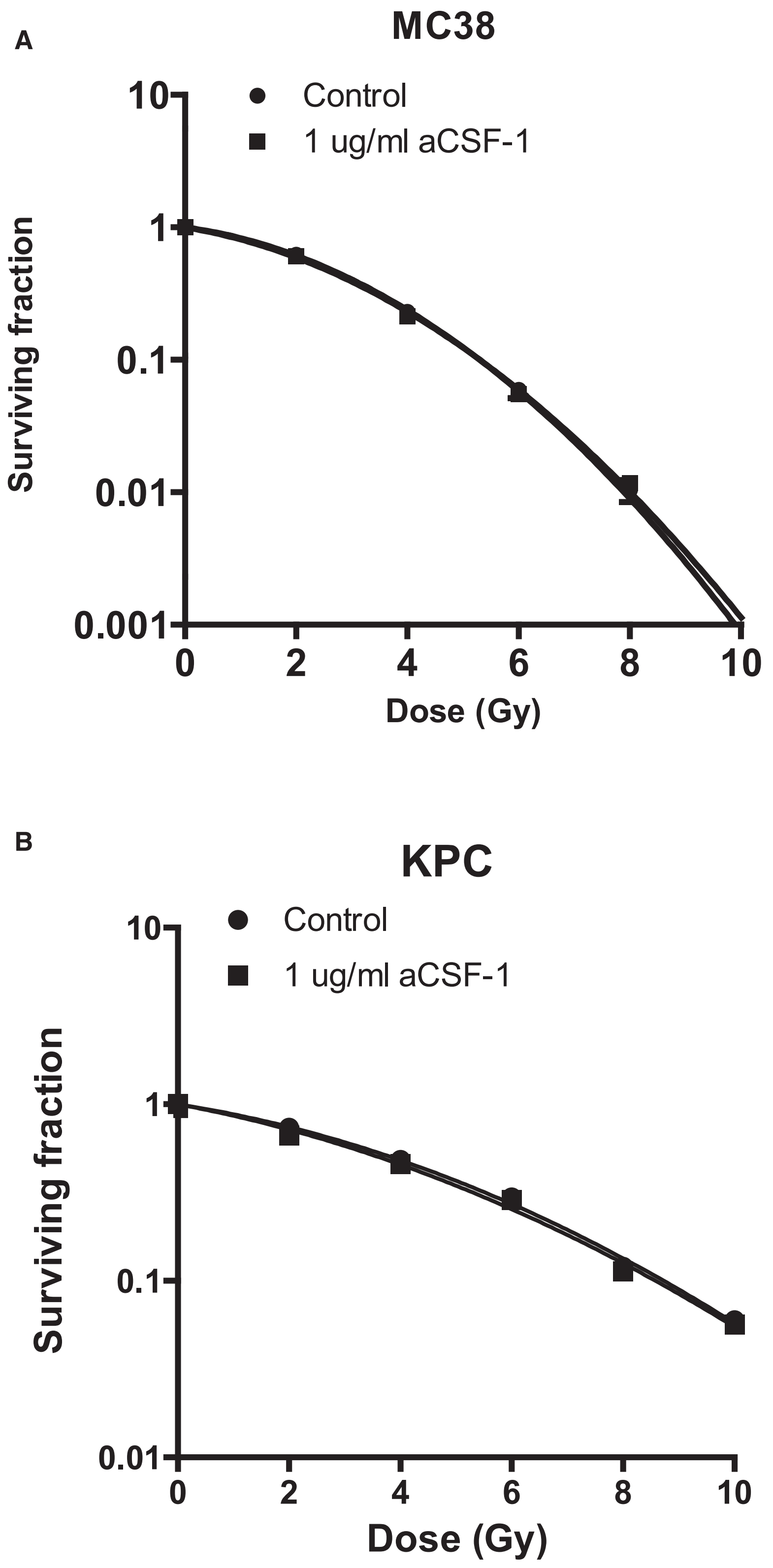

Figure EV3. Anti-CSF does not increase radiosensitivity of MC38 or KPC tumour cells *in vitro*.

A, B Clonogenic survival assays using MC38 (A) and KPC (B) tumour cells with the addition of aCSF. Cells were irradiated using a ^{127}Cs laboratory irradiator to generate doses of 0, 2, 4, 6, 8 and 10 Gy, then cultured for 5–8 days and monitored for colony formation. Colony counting was performed on an Oxford Optronix™ GelCount system using the corresponding software. Comparison of survival between groups for each indicated tie point was analysed by unpaired *t*-test. Data are presented as mean ± SD (*n* = 3/group).

© 2018 The Authors

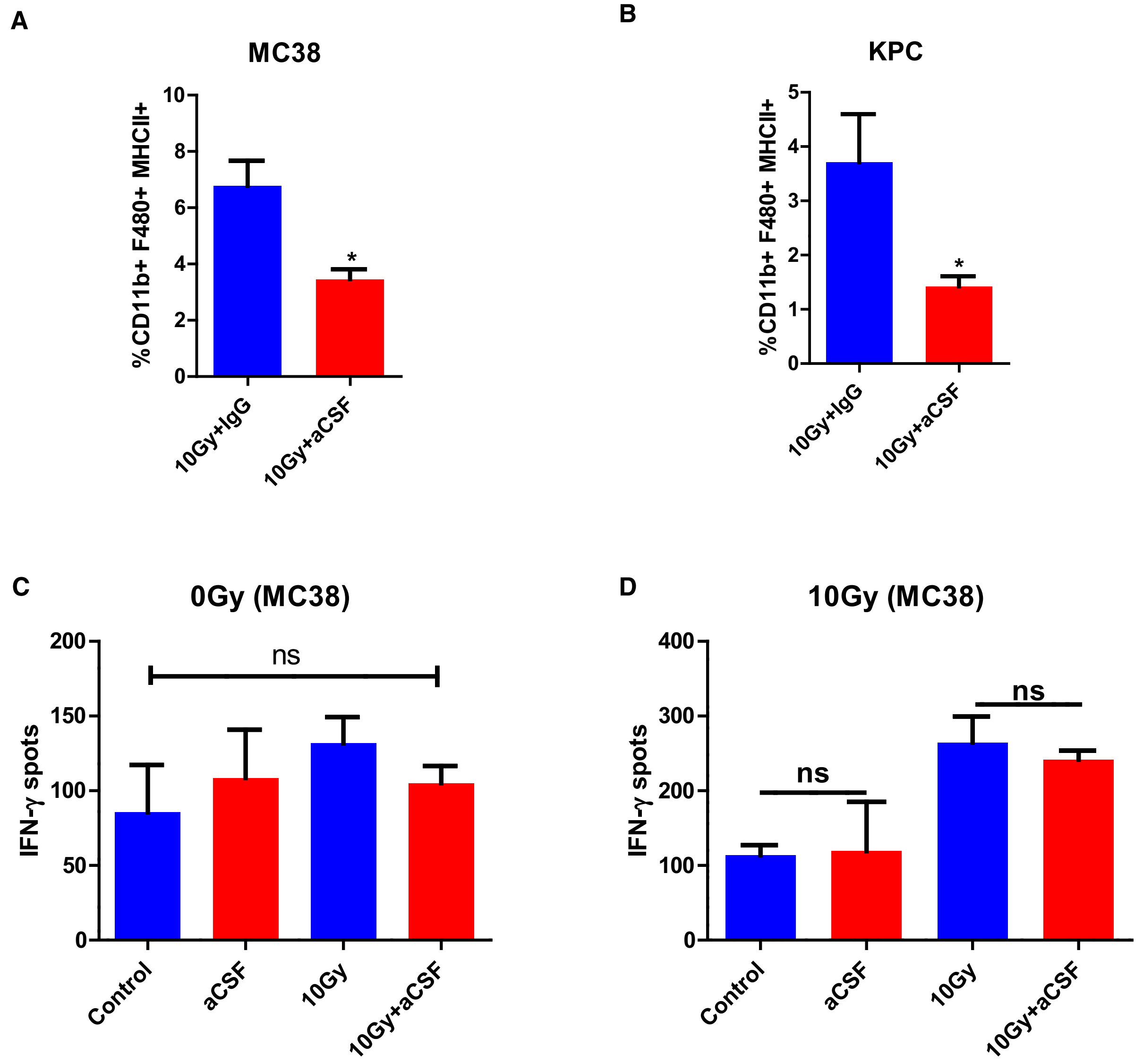

Figure EV4. MHCII$^+$ TAMs are reduced following aCSF treatment, with no effect on systemic T-cell priming.

A, B Flow cytometric evaluation of MHCII$^+$ TAMs (CD11b$^+$F480$^+$MHCII$^+$) in MC38 (A) and KPC (B) tumours 5 days following irradiation. Data shown are mean + SEM and analysed by unpaired *t*-test (n = 3/group). $*P < 0.05$.

C, D Splenic CD8 T cells were isolated from mice bearing MC38 tumours receiving treatment as indicated. T cells were cultured with naïve (C) or irradiated (10 Gy, D) tumour cells for 24 h before IFN-γ ELISpot quantification. Data are presented as mean ± SEM and analysed by Kruskal–Wallis test with Dunn's multiple comparisons test (n = 3 mice/group).

© 2018 The Authors

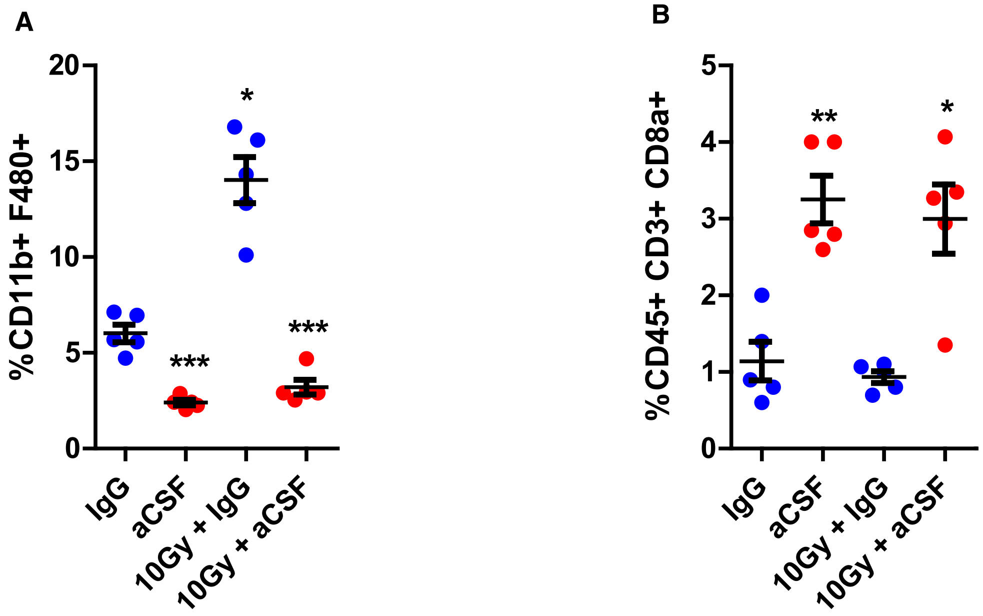

Figure EV5. Macrophage and CD8 populations respond consistently to irradiation in mice bearing two tumours.

A Flow cytometric evaluation of TAMs ($CD11b^{+}F480^{+}$) in primary tumours harvested from mice bearing two MC38 tumours.

B Flow cytometric evaluation of CD8 T cells ($CD45^{+}CD3^{+}CD8a^{+}$) in primary tumours harvested from mice bearing two MC38 tumours.

Data information: Data are presented as mean + SEM and analysed by Kruskal–Wallis test with Dunn's multiple comparisons test (n = 5 mice/group). *$P < 0.05$, **$P < 0.01$, ***$P < 0.001$.

© 2018 The Authors
